# The Effect of Photoluminescence of Bioceramic Irradiation on Middle Cerebral Arterial Occlusion in Rats

**DOI:** 10.1155/2016/7230962

**Published:** 2016-06-07

**Authors:** Lei Zhang, Paul Chan, Zhong-Min Liu, Ling-Ling Hwang, Kuo-Chi Lin, Wing P. Chan, Ting-Kai Leung, Cheuk Sing Choy

**Affiliations:** ^1^Department of Radiology, Shanghai East Hospital, Tongji University, 1800 Yantai Road, Shanghai 200123, China; ^2^Division of Cardiology, Department of Internal Medicine, Wan Fang Hospital, Taipei Medical University, No. 111, Sec. 3, Singlong Road, Taipei 116, Taiwan; ^3^Department of Cardiac Surgery, Shanghai East Hospital, Tongji University, 1800 Yantai Road, Shanghai 200123, China; ^4^Department of Physiology, Taipei Medical University, No. 250, Wu Hsing Street, Taipei 110, Taiwan; ^5^Department of Diagnostic Radiology, Cathay General Hospital, 280 Renai Road, Sec. 4, Taipei 106, Taiwan; ^6^Department of Radiology, Wan Fang Hospital, Taipei Medical University, No. 111, Sec. 3, Singlong Road, Taipei 116, Taiwan; ^7^Graduate Institute of Biomedical Materials and Tissue Engineering, College of Medical Engineering, Taipei Medical University, No. 250, Wu Hsing Street, Taipei 110, Taiwan; ^8^Department of Radiology, Taipei Hospital, Ministry of Health and Welfare, No. 127 Su Yuan Road, Hsinchuang District, New Taipei City 242-13, Taiwan; ^9^Department of Emergency, Min-Sheng General Hospital, 168 Ching-Kuo Road, Taoyuan 330, Taiwan

## Abstract

The purpose of this study is to determine the possible effect of photoluminescence of bioceramic (PLB) on ischemic cerebral infarction (stroke), by using an animal model of transient middle cerebral artery occlusion (MCAO). Sprague-Dawley rats were used to induce MCAO to block the origin of the left MCAO; three months later, the positive chronic stroke rats were selected by running tunnel maze; the MCAO rats with significant chronic stroke and neurological defects were used for treadmill experiments with varying speed settings to test their capability for restoration after muscular fatigue under conditions of with and without PLB irradiation. As a result, PLB irradiation could improve exercise completion rate and average running speed during slow and fast treadmill settings. After PLB irradiation, the selected MCAO rats successfully completed all the second-round treadmill exercises at the maximum speed setting, and they had better restoration from muscular fatigue. An in vitro cell study on astrocytes of rats by bioceramic irradiation further demonstrated increased intracellular nitric oxide. To explain these results, we suggest that cortical brain stimulation of microcirculation and enhancement of peripheral muscular activity are the main causes of the improved exercise performance in MCAO rats by PLB.

## 1. Introduction

Ischemic stroke is the result of reduction in blood flow to the affected brain regions. It causes sudden loss of neurological function after hypoxia of brain tissue and then activates pathogenic cascades after ischemia, disrupting cell metabolism, which eventually results in brain cell death. Ischemic stroke is the biggest cause of physical disability in both developed and developing countries [1, 2]. Different treatments for postischemic stroke to restore normal functioning come at a cost for public healthcare. Basically, the majority of cases with brain damage are not reversible, and recovery of motor function following stroke is limited. It was believed that a possible improvement could happen by functional compensation from residual neural tissue. Providing more effective therapeutic methods and restoring normal function to poststroke patients are still popular research topics in many countries.

Our previous studies showed that bioceramic materials treatment can promote microcirculation [3] and upregulate calcium-dependent nitric oxide and calmodulin [4, 5]. We previously demonstrated that bioceramic materials treatment promotes nitric oxide enhancement through calcium-dependent nitric oxide syntheses [4, 5]. Additionally, bioceramic materials treatment had demonstrated antioxidant effects by increasing hydrogen peroxide scavenging processes in RAW264.7 murine macrophages [6, 7], MC3T3-E1 murine calvaria-derived osteoblast-like cells [8], and C2C12 murine myoblasts [9]. There are beneficial effects of bioceramic irradiation treatment on exercise performance, achieved by decreasing the amount of lactate dehydrogenase released, decreasing metabolic acid accumulation, decreasing tiredness, reducing skin temperature, and stabilizing respiration and heart rates during exercise [9, 10]. The findings were also further proved by in vivo studies on antioxidative stress effects on myoblast cells and preventing fatigue on amphibian skeletal muscles during continuous stimulation [9, 10].

Bioceramic irradiation treatment has also been shown to activate the parasympathetic nervous system, which may improve the recovery of resting cardiac and respiratory rates following submaximal exercise [11]. Additionally, it also contributes to other biological, chemical, and physical properties and has other clinical applications [12–20].

**Figure 1:**
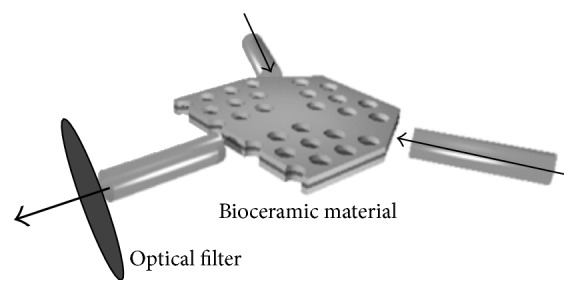
Conceptual picture of PLB, a combination of bioceramic material and visible light spectrum.

Recently, we presented a series of physical-biological experiments on the material based on the characteristics of biological effects of electromagnetic nonionizing radiation [21], nonlinear photonic crystal [22], and photoluminescence [23, 24]. The concept of the “photoluminescence effect” is combining the effects of bioceramic materials, a kind of photonic crystal that emits high-performance ranges of infrared rays, and the visible light spectrum (Figure 1). The characteristics of bioceramic materials may belong to the fifth force field, which belongs to a new concept of physics different from the other four force fields [25]. We attempt to show the possible beneficial effects of PLB on rehabilitation in postinfarcted rats, selected and assessed by their maze performance. Since maze experiments and treadmill exercise are common methods in evaluating postinfarcted rats' performance [26, 27], we adopted these methods to select rats with significant cerebral injuries and to assess the possible recovery of cerebral function under PLB treatment. We also studied the PLB effect by using carotid Doppler ultrasound measurement. The purpose of this study was to evaluate the effects of PLB on stroke, using animal studies after postischemic infarction.

## 2. Materials and Methods

The ceramic powder was obtained from the Laboratory of Radiology in Taipei Hospital (New Taipei City, Taiwan). The bioceramic material consisted of microsized particles produced primarily from different elemental components.

### 2.1. Photoluminescence of Bioceramic Materials (PLB)

The concept and procedure of PLB irradiation involve the application of specially manufactured bioceramic materials that consists of microsized particles produced from various elemental components [4–20]. Of the bioceramic material, seven percent was embedded in a silicon sticker with good translucence (Bioenergy Development Ltd., Taoyuan, Taiwan). Photoluminescence is a special type of luminescence, referring to materials that absorb light energy and then release that energy in the form of light; it also describes the interaction between electromagnetic (EM) radiation and matter. The PLB of this bioceramic material absorbs a portion of the wavelength of the EM spectrum that includes near-, middle, and far infrared rays. The concept of PLB is a visible light source of light-emitting diodes (LEDs) without additional thermal effect, propagated through bioceramic materials for irradiation on living animals (Figure 1).

### 2.2. Animal Model Experiments

Sprague-Dawley rats (200 g; *n* = 10) were maintained in cages at 23°C ± 2°C and 60%  ±  5% humidity with 12-hour light/dark cycling. Solid rat food and water were continuously available during the first week to allow the rats to adjust to the environment. All procedures were conducted in accordance with the ethical guidelines for animal experiments established by our institution (IACVC approval number: LAC-101-0093). Anesthesia was induced with chloral hydrate dissolved in normal saline (35 mg/100 gm, intraperitoneal) and was maintained during the procedure by supplemental doses as needed. Body temperature was monitored with a rectal thermometer and was maintained within normal limits with a heating pad.

**Figure 2:**
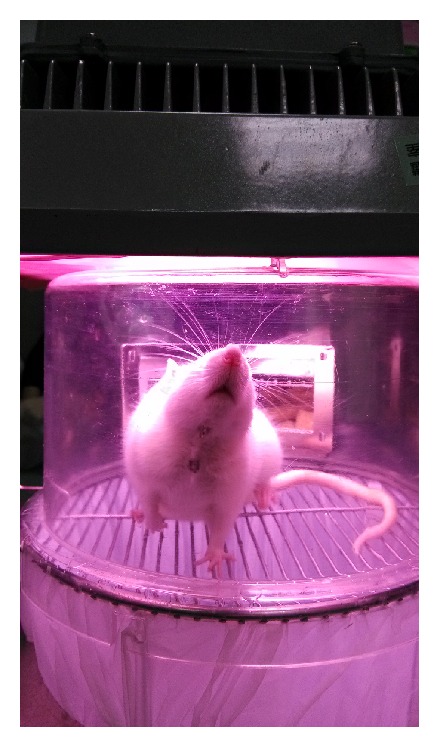
PLB irradiation on MCAO rats with surgical clips.

Transient MCAO was induced by advancing a 4-0 surgical nylon suture into the internal carotid artery (ICA) to block the origin of the MCA. A length of 18.5 to 19.5 mm 4-0 surgical nylon suture, determined by animal weight, was advanced from the left external carotid artery (ECA) into the lumen of the ICA until it blocked the origin of the MCA. The suture was withdrawn to restore blood flow and reperfusion after two hours and then the wound was clipped. As mentioned, selected rats were irradiated using a light source of combining LED (wavelength of visible light, as determined by spectroscopy by the Taiwan Textile Research Institute) and a photonic crystal of bioceramic material. The light sources were kept 10 cm from the rats' bodies (Figure 2). We also controlled the light illumination measured by illuminometer, constantly at 450 ± 50 lux, and irradiation for four hours as one section.

### 2.3. Assessment and Selection of MCAO Rats for PLB Treatment

To assess the amount of cerebral damage three months after the MCAO procedure (to be defined as chronic stroke status), we used a simple tunnel maze (60 × 45 × 60 cm), made of nontransparent material. Before a real test was performed, each rat received five practice runs in the tunnel maze, so as to develop experience and prevent fearfulness.

We selected the MCAO rats that expressed poor maze performance, those finishing the tunnel maze in over 200 seconds, to continue our tests.

### 2.4. Selected MCAO Rats for Tunnel Maze Experiment under PLB Treatment

The selected MCAO rats ran the tunnel maze without PLB and then received PLB treatment on another day at the same time, 2:00 p.m., so as to prevent uncontrollable factors on the levels of adrenaline and metabolic rates. The MCAO rats were kept in the tunnel maze for 200 seconds and 30-minute PLB treatments were then provided for each rat, and their subsequent tunnel maze performance was used for comparison.

### 2.5. Selected MCAO Rats for Treadmill Experiment under PLB Irradiation

**Figure 3:**
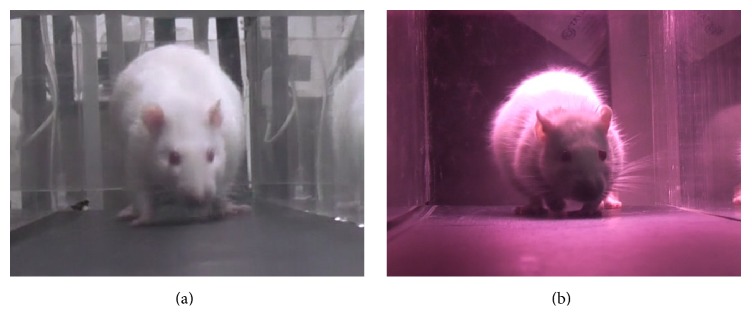
Running rat without PLB irradiation (a) and with PLB irradiation (b).

MCAO rats underwent the treadmill experiment at different speed settings of 10 cm/s, 20 cm/s, 30 cm/s, 40 cm/s, 50 cm/s, 60 cm/s, high speed (80 cm/s), and maximum speed (100 cm/s). From start to finish, the rats would complete three rounds of running at each speed setting. To be successful, the rat had to complete the entire distance; inability to complete the entire distance would be regarded as “failure” (Figure 3). Time required to finish the distance from the starting point to the finishing point was recorded for each MCAO rat. The distances of running speed per second were also calculated. We compared the control group (without PLB) and the experimental group (with PLB) to investigate whether the running velocity of MCAO rats could be improved.

### 2.6. PLB Irradiation on Selected MCAO Rats to Assess the Ability to Restore Muscular Fatigue

In the beginning, the selected MCAO rats finished the treadmill running and rested for 30 minutes without PLB irradiation. They were then divided into control and experimental (PLB irradiation) groups. The second round of treadmill running was designed with different treadmill speed settings to assess whether PLB irradiation could restore muscle fatigue on the selected MCAO rats.

### 2.7. Selected MCAO Rats for Carotid Doppler Ultrasound Experiment under PLB Irradiation

**Figure 4:**
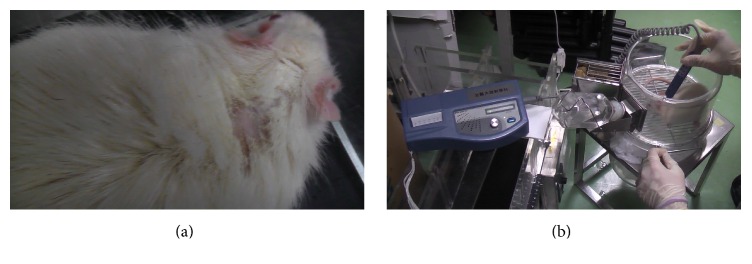
Hair shaved rats on the dorsal neck; (a) Doppler ultrasound measurement performed on carotid blood flow (b).

In this experiment, a Doppler ultrasound blood flow meter (BV 520, LG Biotech, Shanghai, China) was used to measure the MCAO rat carotid artery blood flow, including mean arterial blood flow velocity measurements (MN) and the maximum spectral peak (PK). First, the selected MCAO rats' hair was shaved from the dorsal neck, and then the Doppler ultrasound on carotid blood flow (DUCBF) was measured and recorded for PK and MN (Figure 4). For each selected MCAO rat, the DUCBF was measured four times: (1) before treadmill exercise; (2) after treadmill exercise; (3) after 30 minutes of rest with PLB irradiation before the second round of treadmill exercise; and (4) shortly after the second round of treadmill exercise.

### 2.8. Measurement of Nitric Oxide (NO) Production on Primary Cell Culture of Astrocyte with and without PLB Irradiation

Primary cultures of rat cortical neurons (provided by Professor Yi Hsuan Lee of the Department of Physiology, Taipei Medical University, Taiwan) were prepared from the cerebral cortex of 14-day-old rat foetuses. The cerebral cortices of foetuses obtained under sterile conditions were dissected and dissociated mechanically, by pipetting 10 times with 10 mL of DMEM (Gibco Invitrogen Corporation, Barcelona, Spain). The cell suspension was filtered through nylon mesh with a pore size of 90 *μ*m. Cell suspension was plated (5 × 10^4^ cells/cm^2^) on polylysine coated dishes. After attachment of the cells, the plating medium was changed to DMEM containing 10% FBS supplemented with antibiotics (1%) and fungizone (1%). Cultures were grown in a humidified atmosphere of 5% CO_2_/95% air at 37°C for 3 days. Cells were then exposed to 10 *μ*M cytosine *β*-D-arabinofuranoside on the third day of culture for 24 h to inhibit proliferation of nonneuronal cells. The medium was changed twice a week.

**Figure 5:**
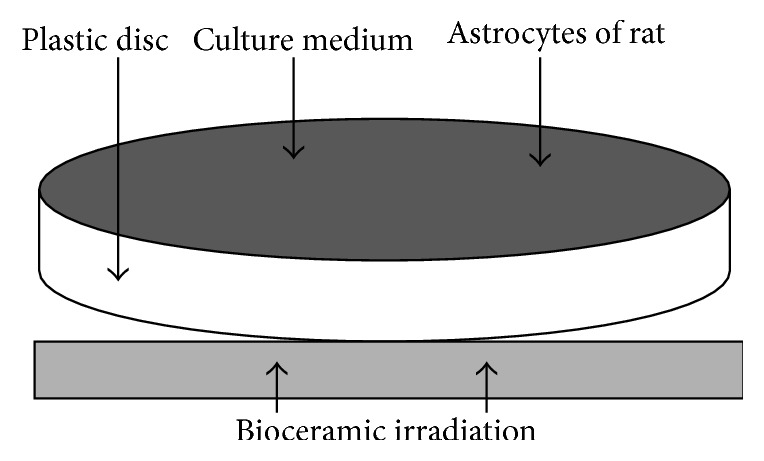
In vivo experimental model of bioceramic irradiation on astrocytes of rats, in addition to NO concentration measurement.

The cells are separated as control and bioceramic irradiation groups, while the bioceramic irradiation group received bioceramic irradiation for 10 minutes. The cells and source of the bioceramic irradiation were separated by plastic culture discs, without direct contact (Figure 5), similar to MACO rats' brain tissue indirectly exposed from PLB.

All cell dishes were then stained with DAF-FM diacetate for fluorescence measurements for NO. All cells were analyzed by a fluorescence-activated cell sorter (FACS) and flow cytometry at the single-cell level. All the data were acquired and analyzed, and the mean fluorescence intensities of intracellular NO production in astrocytes of rats were determined.

### 2.9. Data Analysis

All experiments were done at least three times, each time with two or more independent observations. Statistical analysis was performed by paired *t*-test.

## 3. Results

### 3.1. Number of MCAO Rats Selected for PLB Treatment

**Figure 6:**
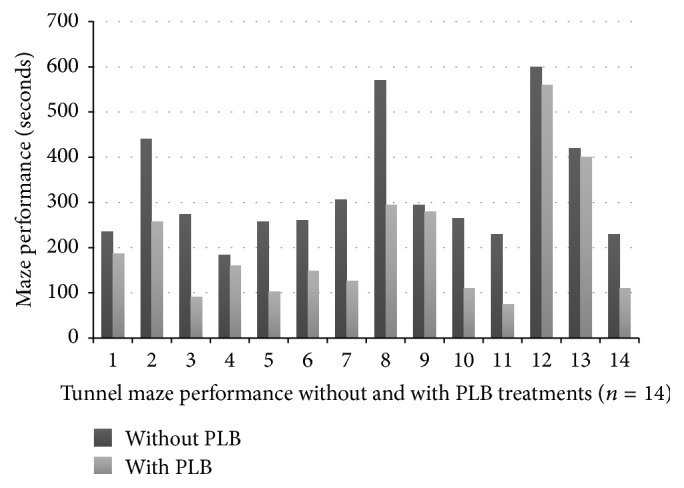
Comparison of MCAO rats on tunnel maze performance, with and without PLB.

After the MCAO procedure on ten rats, three of the rats expired within 24 hours of the surgery. Although the surviving rats initially expressed transient neurological deficits on motor behavior, all of the MCAO rats were nearly recovered in general appearance. In order to select the brain damaged rats after the MCAO procedure, the rats underwent tunnel maze tests. Three out of the seven rats exhibited poor performance on the tunnel maze tests, taking more than 200 seconds, and were selected for PLB treatments (Figure 6). This indicates that the selected MCAO rats had confirmed significant neurological defects.

### 3.2. PLB Treatment Reduces the Time Period Needed to Complete the Tunnel Maze in Selected MCAO Rats

Our data showed improvements in the required time for the three selected MCAO rats (number 3, number 4, and number 8) to complete the tunnel maze after PLB treatments (*n* = 14), which is a statistically significant difference (*p* < 0.05). Once the PLB treatment was provided for the selected MCAO rats, it was necessary to wait for two to three days until the disappearance of previous bioceramic irradiation effects.

### 3.3. Results of PLB Treatment on Selected MCAO Rats for Treadmill Running

**Figure 7:**
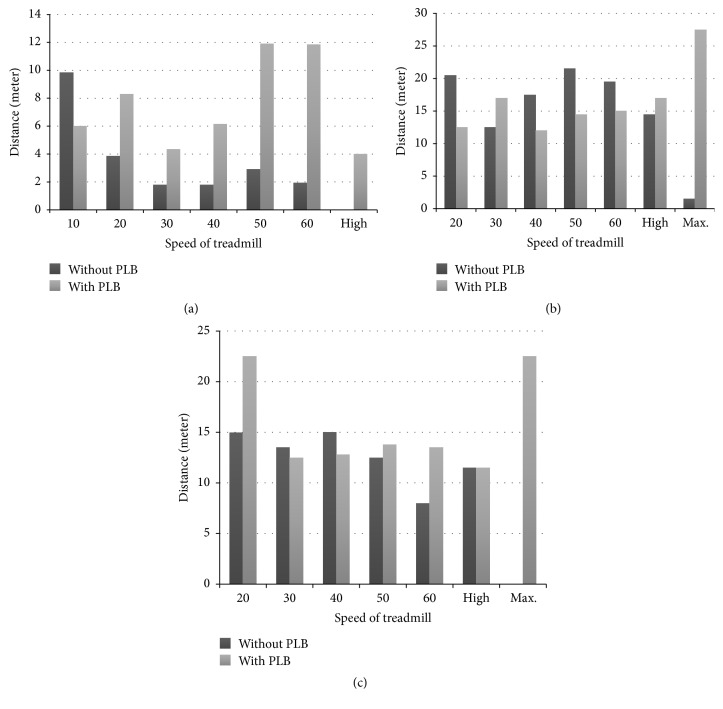
(a) The performance of number 3 rat with and without PLB, during running distance (meter) on treadmill under different speeds (cm/sec). (b) The performance of number 4 rat with and without PLB, during running distance (meter) on treadmill under different speeds (cm/sec). (c) The performance of number 8 rat with and without PLB, during running distance (meter) on treadmill under different speeds (cm/sec).

Based on statistical results of the selected MCAO rats from the second round of treadmill exercise to assess muscular fatigue restoration, it was found that PLB irradiation could enhance the completion rate and average speed (Figures 7(a)–7(c)). For example, number 3 rat failed to finish an additional treadmill exercise without PLB irradiation and expressed exhaustion. It ran slowly and was then being dragged by the running treadmill at the end of the exercise. On the contrary, PLB irradiation promotes completion rate, faster running, and better achievements on all treadmill speed settings. Particularly, better performance after PLB irradiation on the second round of treadmill exercise was very significant on high-speed running. The average rate of running is 4.07 ± 6.65 cm/s. The results of number 4 and number 8 MCAO rats on the second round of treadmill exercises showed only mild improvement on their performance differences with PLB irradiation and without PLB irradiation. However, at maximum speed, there were significant differences; the average rate is 27.47 ± 0.84 for the PLB irradiation group and 22.54 ± 19.91 for the control group (*p* < 0.05).

### 3.4. Results of PLB Treatment on Selected MCAO Rats for Restoration of Muscular Fatigue

**Figure 8:**
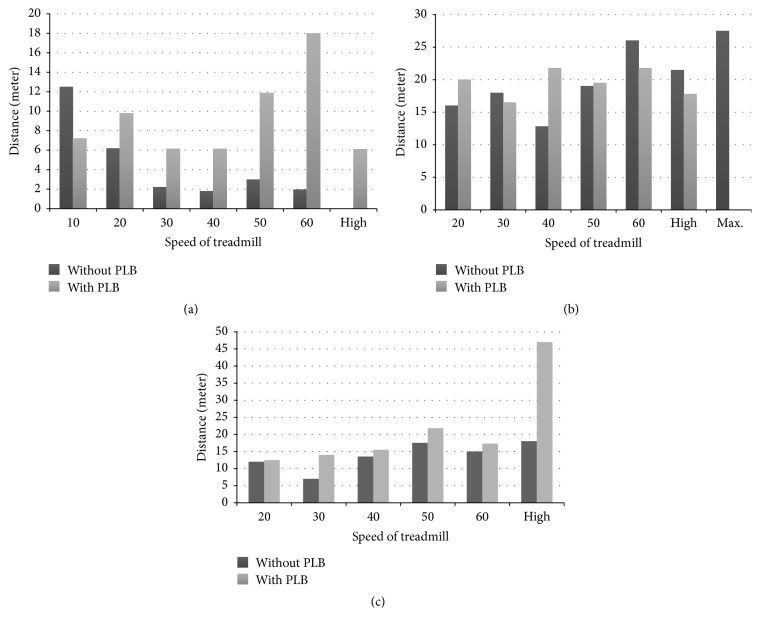
(a) The performance of number 3 rat with and without PLB for restoration of muscular fatigue, by running distance (meter) on treadmill under different speeds (cm/sec). (b) The performance of number 4 rat with and without PLB for restoration of muscular fatigue, by running distance (meter) on treadmill under different speeds (cm/sec). (c) The performance of number 8 rat with and without PLB for restoration of muscular fatigue, by running distance (meter) on treadmill under different speeds (cm/sec).

In the beginning, the MCAO rats failed to finish the exercise at the maximum speed setting. After PLB irradiation, it was found that number 3, number 4, and number 8 MCAO rats completed all the second-round treadmill exercises at the maximum speed setting by an average speeds rate of 6.11 ± 7.34 cm/s (number 3), 27.47 ± 0.84 cm/s (number 4), and 47 ± 2.48 cm/s (number 8) (Figures 8(a)–8(c)). Comparing the control and PLB irradiation groups shows a significant difference (*p* < 0.05).

### 3.5. Results of PLB Treatment on Parameters of Carotid Doppler Ultrasound

In the beginning, we predicted significant differences of MN and PK by DUCBF measurement of PLB irradiation before and after treadmill exercise. In comparing PLB irradiation before and after treadmill exercise, however, the results of DUCBF measurements showed no significant differences on PK and MN before and after PLB irradiation with *p* > 0.05 (Table 1).

### 3.6. Results of Nitric Oxide Production on Astrocytes of Rats under PLB Irradiation

**Figure 9:**
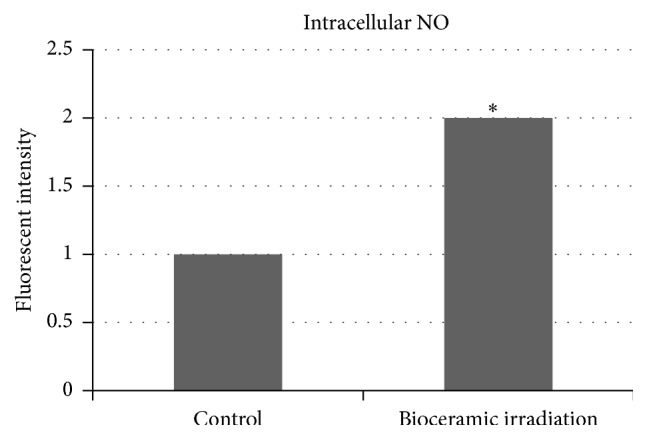
By cytometric analysis, NO production in bioceramic irradiation group within astrocytes of rats is statistically significantly increased (^*∗*^ *p* < 0.05) compared with the control group.

Levels of intracellular NO synthesis of astrocytes of rats in the control and bioceramic irradiation groups are shown in Figure 9. The *p* value between these two groups is less than 0.05, which shows a significant difference.

## 4. Discussion

Stroke is a leading cause of adult motor disability, and the corresponding recovery of motor function from stroke is usually a slow and frustrating process [26]. From our results, both maze performance and treadmill exercise could be improved by using PLB irradiation. In our opinion, cortical brain stimulation of microcirculation and enhancement of peripheral muscular activity are the main causes for improved performance in MCAO rats undergoing PLB. Before we discuss the possible mechanisms of how PLB irradiation affects the behavior of MCAO rats, we should review clinical methods that may improve poststroke or brain cortex ischemic injury of humans and rats. For example, clinical uses of repetitive transcranial electrical and magnetic stimulations were developed to improve brain activity after ischemic stroke [27]. For magnetic stimulation, a pulsed magnetic field creates current flow in the brain and can temporarily excite or inhibit specific areas. Transcranial magnetic stimulation of the motor cortex can produce muscle twitch or block movement; transcranial magnetic stimulation of occipital cortex can alter the brain function beyond the time of stimulation, offering potential for therapy. Electrical and magnetic methods are available to stimulate the human brain through the intact scalp. The effects of these less invasive cortical stimulations on the extended time course of learning have beneficial and promising implications on motor learning protocols in healthy individuals and in stroke patients undergoing neurorehabilitation [28, 29]. However, the mechanisms of these two methods are not well understood and not widely used clinically. On the other hand, hyperbaric oxygen (HBO) therapy was also proven to have the capability to reduce ischemic brain damage and behavioral dysfunctions, which may reduce the infarct area and improve neurologic scores at seven days after reperfusion [28–31]. One of the molecular mechanisms of HBO-induced brain protection is preventing apoptosis, and this effect of HBO might preserve more brain tissues and promote neurologic functional recovery. HBO can reduce brain tissue apoptosis, especially when applied after the initial ischemia; HBO might be used as an alternative or additional therapy for patients who have had an acute stroke because no effective treatment is available [30, 31]. The effects of reduced behavioral dysfunctions of ischemic stroke for both animals and patients by HBO therapy are related to upregulating nitric oxide that promotes microcirculation and helps recover damaged brain tissue, probably in acute and subacute stages [32–35]. However, there is still not enough quantitative measurement to determine the effectiveness of HBO treatment on recovery of chronic stroke patients [32].

In this study, PLB irradiation externally on chronic stroke rats is a new kind of cortical stimulation, without applying magnetism and strong electricity. We had previously demonstrated that bioceramic materials treatment was able to promote microcirculation in patients [3] by upregulating calcium-dependent nitric oxide and calmodulin in cell lines [4, 5]. In this present study, we demonstrate an in vitro study on astrocytes of rats, with a statistically significant increase of NO production by bioceramic irradiation. Increasing the intracranial microcirculation by bioceramic irradiation is a kind of brain stimulation, acting as one of the reasons to explain how PLB improved exercise performance of MCAO rats on both maze and treadmill tests. Apart from brain stimulation, practice of rehabilitation on stroke patients is also achieved by strengthening peripheral muscles. Clinically, postinfarcted patients are trained for different walking speeds and inclinations on the treadmill platform, to provide treatment on different paretic and nonparetic sides of muscles [36]. For example, methods to improve medial gastrocnemius activity of poststroke patients may improve their treadmill performance [36]. One of our previous studies showed the effects of bioceramic materials on oxidative stressed murine myoblast cells (C2C12) and fatigue conditions of amphibian skeletal muscle during exercise [9]; this helped to explain PLB treatment on MCAO rats for restoration of muscular fatigue. We had demonstrated that bioceramic irradiation received beneficial effects on viability of H_2_O_2_-mediated oxidative stress induced C2C12 cells and decreasing lactate dehydrogenase release and resulted with reduction of muscular fatigue [9]. It was proven that nitric oxide (NO) within C2C12 cell was elevated after bioceramic irradiation. We had further used a physiological experimental model of electrostimulation on amphibian skeletal muscle, stimulating the muscle samples by continuous pulses for a long period of time and keeping record of contraction loading force until onset of muscle fatigue. In comparing the mean contraction force between the bioceramic irradiation group and control group, there was a significant difference (*p* < 0.05) between the two groups on the mean contraction loading forces, which indicates that bioceramic irradiation successfully reduced fatigue in amphibian muscles. Furthermore, measurement of pH changes on muscles after fatigue was done after the electrostimulated isometric contractions associated with bioceramic irradiation. The results showed a decrease on metabolic acid accumulation with measurable elevation of pH value on the muscular tissue, as compared to the control group. Thus, bioceramic irradiation can normalize acidification following muscle contractions and delay onset of muscular fatigue [9]. Additionally, a human trial of strenuous exercise in a model of treadmill running was performed with and without bioceramic irradiation, recorded with tendencies of decreased tiredness and reduced skin temperature in the bioceramic irradiation group. Moreover, more stable respiration rate and heart rate on candidates were recorded in the bioceramic irradiation group compared with the control group [10]. One of the limitations in this study is that the Doppler ultrasound measurement on MCAO rats of post-PLB irradiation was not able to determine its effectiveness on PK and MN. The reason is the technical difficulties in data collection on uncontrollable and moving rats. In the future, additional experiments or new methods will be required to approach PLB effect on carotid blood flow.

## 5. Conclusion

We found that PLB irradiation was able to improve different running speeds of the selected MCAO rats, particularly in maximum speed treadmill running and second-round exercise. We suggest that cortical brain stimulation of microcirculation and enhancement of peripheral muscular activity are the two main causes for better exercise performance in MCAO rats by PLB. In the future, medical instrument development for clinical PLB trials is necessary for chronic cerebral infarction. The above findings can be of important reference for future medical device development. Further research of human trial on the correlations among regional cerebral flow, neuromuscular performance, and motor restoration is necessary.

## Figures and Tables

**Table 1:** Results of selected MCAO rats for carotid Doppler ultrasound experiment with and without PLB irradiation (*p* value > 0.05; experimental data exhibit no significant difference).

Number of MCAO rat	Timing of DUCBF measurement	DUCBF measurement without PLB	DUCBF measurement with PLB	Data subtraction of measurement without PLB from that with PLB
Number 3	Preexercise PK	0.378	0.284	0.094
	Postexercise PK	0.336	0.473	−0.137
	Preexercise MN	0.176	0.162	0.014
	Postexercise MN	0.183	0.217	−0.034

Number 4	Preexercise PK	0.432	0.444	−0.012
	Postexercise PK	0.84	0.835	0.005
	Preexercise MN	0.217	0.254	−0.037
	Postexercise MN	0.34	0.59	−0.25

Number 8	Preexercise PK	0.434	0.738	−0.304
	Postexercise PK	1.163	0.7	0.463
	Preexercise MN	0.296	0.538	−0.242
	Postexercise MN	0.75	0.573	0.177

MN: mean arterial blood flow velocity measurements; PK: maximum spectral peak.
